# Influence of the gestational stage on the clinical course, lesional development and parasite distribution in experimental ovine neosporosis

**DOI:** 10.1186/s13567-014-0139-y

**Published:** 2015-03-03

**Authors:** David Arranz-Solís, Julio Benavides, Javier Regidor-Cerrillo, Miguel Fuertes, Ignacio Ferre, Maria del Carmen Ferreras, Esther Collantes-Fernández, Andrew Hemphill, Valentín Pérez, Luis Miguel Ortega-Mora

**Affiliations:** SALUVET, Animal Health Department, Faculty of Veterinary Sciences, Complutense University of Madrid, Ciudad Universitaria s/n, 28040 Madrid, Spain; Livestock Health and Production Institute (ULE-CSIC), 24346 León, Spain; Institute of Parasitology, Vetsuisse Faculty, University of Bern, CH-3012 Bern, Switzerland

## Abstract

*Neospora caninum* is considered one of the main causes of abortion in cattle, yet recent studies have also emphasised its relevance as an abortifacient in small ruminants. In order to gain deeper insight into the pathogenesis of ovine neosporosis, pregnant ewes were intravenously inoculated with 10^6^ tachyzoites of the Nc-Spain7 isolate at days 40, 90 or 120 of gestation. Infection during the first term resulted in the death of all foetuses between days 19 and 21 post-infection, showing mainly necrotic lesions in foetal liver and the highest parasite DNA detection and burden in both placenta and foetal viscera. After infection at day 90, foetal death was also detected in all ewes, although later (34–48 days post-infection). In this group, lesions were mainly inflammatory. Foetal livers showed the lowest frequency of lesions, as well as the lowest parasite detection and burden. All ewes infected at day 120 delivered viable lambs, although 3 out of 9 showed weakness and recumbency. *Neospora* DNA was detected in all lambs but one, and parasite burden was similar to that observed in day 90 group. Lesions in this group showed more conspicuous infiltration of inflammatory cells and higher frequency in foetal brain and muscle when compared to both previous groups. These results highlight the crucial role that the stage of gestation plays on the course of ovine neosporosis, similar to that reported in bovine neosporosis, and open the doors to consider sheep as a valid model for exogenous transplacental transmission for ruminant neosporosis.

**Electronic supplementary material:**

The online version of this article (doi:10.1186/s13567-014-0139-y) contains supplementary material, which is available to authorized users.

## Introduction

*Neospora caninum* (Apicomplexa: Eimeriina: Sarcocystidae) is an obligate intracellular parasite, which is regarded as one of the most important infectious causes of abortion in cattle worldwide [1]. Since its discovery, *N. caninum* has been identified in various species of livestock, including cattle, sheep, goats, horses and deer [2]. Cattle can become infected by horizontal transmission via the ingestion of oocysts, or by vertical transmission (i.e. transplacentally) as a result of either a primary infection of the dam by oocysts (exogenous transplacental transmission) or recrudescence of a chronic infection (endogenous transplacental transmission) during pregnancy, with different clinical and epidemiological consequences [3].

Although cattle represent the most relevant and economically important target host, the presence of *Neospora* infection and transplacental transmission in small ruminants have been previously reported. In sheep, naturally occurring *N. caninum* transmission was first described in a congenitally infected lamb [4]. Subsequently *N. caninum* infections in sheep have been reported worldwide [5-8]. Nevertheless, *Toxoplasma gondii* is regarded as the primary parasite cause for both sheep and goat abortion [1,6,8], and the epidemiological, clinical and economic importance of neosporosis in small ruminants has been traditionally considered to be much less relevant compared to cattle [9,10]. However, recently acquired evidence suggests that *N. caninum* is an important abortifacient in small ruminants [6], or even the main cause of reproductive losses in some flocks [7,8]. Despite the obvious significance of ruminant neosporosis, there are still many aspects that require further studies, such as host-pathogen interactions, pathogenicity and immunological control. Previous experimental infections in pregnant ewes have shown that sheep are highly susceptible to infection, and display a similar pathogenesis and disease outcomes as reported for cattle [9,11,12]. It has been suggested that the time point of infection during gestation plays a key role in the pathogenesis of the disease [9,11-16]. However, it is complicated to compare the results from these studies, as they differ in experimental design, parasite isolate and infection dose, route of administration and time point of infection. Moreover, there are not reports on the outcomes of infection during different pregnancy periods within the same study and under the same experimental conditions. Clearly, the availability of well-defined ruminant infection models is a key factor for the evaluation of vaccine and drug candidates [17]. As an experimental animal model, sheep exhibit several advantages over cattle, including size, length of gestation and cost. Therefore, the aim of this study was to investigate the outcome of experimental infection by *N. caninum* in ewes under standardized conditions at an early, mid-term and late period of gestation. This allowed to evaluate the effect of the gestation period on the clinical course of disease, lesion development and parasite distribution in different organs, and to assess the validity of experimental infection in sheep as a model for exogenous transplacental transmission for ruminant neosporosis.

## Material and methods

### Ethics statement

All protocols involving animals were approved by the Animal Welfare Committee of the Livestock Health and Production Institute (IGM, ULE-CSIC), León, Spain, following proceedings described in Spanish and EU legislations (Law 32/2007, R.D. 1201/2005, and Council Directive 2010/63/EU). All animals used in this study were handled in strict accordance with good clinical practices and all efforts were made to minimize suffering.

### Animals and experimental design

Animals used in this study came from the *Churra* sheep breed flock of ULE-CSIC, which is kept in dedicated animal facilities and free of *N. caninum* infection or any other abortifacient agent. Fifty pure *Churra* breed sheep aged 12 months were selected after checking by ELISA their seronegativity for *T. gondii*, *N. caninum*, Border disease virus (BDV), *Coxiella burnetii* and *Chlamydophila abortus*. These sheep were oestrus synchronized and mated with pure breed *Churra* tups for 2 days, after which the rams were removed from the ewes. Pregnancy and foetal viability were confirmed by ultrasound scanning (US) on day 40 after mating, and twenty-nine pregnant sheep were selected for the experiment.

Pregnant sheep (*n* = 29) were randomly distributed into five experimental groups. Twenty ewes were allocated in groups 1 (G1; *n* = 6), 2 (G2; *n* = 7) and 3 (G3; *n* = 7), which were inoculated intravenously (IV) with 10^6^ culture-derived tachyzoites of the Nc-Spain7 bovine isolate [18] at day 40 (G1), 90 (G2) or 120 (G3) of gestation (dg), respectively. The nine remaining sheep were allocated in groups 4 (G4; *n* = 6) and 5 (G5; *n* = 3) as controls of infection and pregnancy, respectively. Two animals from G4 and one from G5 received an IV inoculum of phosphate-buffered saline (PBS) at each time point of infection (day 40, 90 or 120), thus two negative controls animals and one sentinel control were available for each time point. Ewes from G4 were culled at the average time points when abortion took place in their respective group, providing a negative control for further analyses (see below), and ewes from G5 were kept alive until the end of the experiment (Table 1).

**Table 1:** **Experimental design**

**Group**	**Number of pregnant ewes**	**Number of foetuses/lambs**	**Days of gestation at the time of inoculation**	**Inoculum (IV)**	**Animal euthanasia** ^**a**^
**G1**	6	11	40	Nc-Spain7 10^6^ tachyzoites	Yes
**G2**	7	12	90	Nc-Spain7 10^6^ tachyzoites	Yes
**G3**	7	9	120	Nc-Spain7 10^6^ tachyzoites	Yes
**G4**	6	9	40-90-120^b^	PBS	Yes^b^
**G5**	3	4	40-90-120^c^	PBS	No^c^

IV: intravenous route.

^a^Animals were culled when fœtal death was detected or immediately after parturition.

^b^In G4, 2 ewes correspond to each period of inoculation (40, 90 and 120), and were culled at the average day of abortion of each group (i.e. 20 dpi for G1 and 40 dpi for G2) or at parturition (for G3).

^c^In G5, 1 ewe corresponds to each period of inoculation (40, 90 and 120). All three ewes gave birth to healthy lambs and were kept alive until the end of the experiment.

### Parasites

Tachyzoites of the Nc-Spain7 isolate were routinely maintained in cultured MARC-145 cells as described previously [19]. The experiment was carried out using similar parasite passage numbers in MARC-145 cells cultures for all three days of inoculation (passage No. 12 for G1, No. 8 for G2 and No. 11 for G3). Inocula were prepared as described previously [19]. Briefly, tachyzoites were recovered from culture flasks when they were still largely intracellular and at least 80% of the parasitophorous vacuoles were undisrupted. Tachyzoite numbers were determined by Trypan blue exclusion followed by counting in a Neubauer chamber, and parasites were resuspended in PBS at the required dose of 10^6^ tachyzoites in a final volume of 2 mL. Tachyzoites were administered to ewes within 30 min of harvesting from tissue culture.

### Clinical monitoring and collection of samples

Following experimental infection, ewes were observed daily throughout the entire experimental period. Foetal viability was assessed by the transabdominal ultrasonography (US) monitoring of foetal heartbeat and movements. All ewes were checked upon once weekly for the two first weeks post-infection (pi), and then twice weekly until detection of foetal death. When foetal death occurred, or immediately after parturition, dams and lambs were first sedated with xylazine -Rompun- (Bayer, Mannhein, Germany) and then immediately euthanized by an IV overdose of embutramide and mebezonium iodide -T61- (Intervet, Salamanca, Spain). Animals from G4 were culled at 20 days post-inoculation (dpi) for G1, 40 dpi for G2 and at parturition for G3. Animals from G5 were examined once per month for pregnancy by US from the sixth week until lambing, and ewes were kept alive.

Post-mortem examination of the ewes and lambs was carried out immediately after euthanasia, and foetuses were immediately separated from the placenta. Samples for serological, histological and molecular studies were collected as follows: blood samples were taken on the day of necropsy by jugular vein-puncture in Vacutainer tubes (Becton Dickinson and Company, Plymouth, UK) without anticoagulant and were allowed to clot. Serum was obtained by centrifugation and samples were stored at −80 °C until analysis. After necropsy, five randomly selected placentomes were recovered from each placenta and were transversally cut in slices of 2–3 mm of thickness that were distributed for storage in 10% formalin for histopathological examinations, and the rest of the placentomes were stored at −80 °C for further parasite DNA detection by PCR. Brain (one half at −80 °C and one half in 10% formalin) and iliofemoral (uterine) and mesenteric lymph nodes from dams were collected for PCR and histopathological analysis. Foetal tissues (brain, heart, liver, lungs and a portion of semitendinosus skeletal muscle) were stored at −80 °C for DNA extraction and fixation in 10% formalin. Foetal thoracic and abdominal fluids or precolostral serum were also collected and maintained at −80 °C for serology. To prevent any transmission of colostral antibodies from dams, lambs were separated from their mothers immediately after birth, sampled for blood and euthanized. In order to avoid any accidental suckling from lambs born overnight, udders were covered with a piece of cloth one week before the expected date of delivery as a preventive measure.

### Serological analyses: IFAT

Indirect fluorescent antibody test (IFAT) was used to detect specific IgG anti-*Neospora* antibodies in foetal fluids, precolostral sera, and sera from dams, according to the technique previously described by Álvarez-García et al. [20]. Foetal fluids, precolostral sera and sera from dams were diluted at two-fold serial dilutions in PBS starting at 1:8 (for foetal fluids and precolostral sera) and 1:200 (for sera from dams), up to the end point titre. Unbroken tachyzoite membrane fluorescence at a titre ≥ 8 for foetal fluids or precolostral sera and ≥ 200 for sera from dams was considered a positive reaction.

### Histopathology and lesion scoring

After fixation in formalin for five days, maternal and foetal brains were cut coronally, embedded in paraffin wax, and were processed, with the rest of the samples, by standard procedures for haematoxylin and eosin (HE) staining. Conventional histological evaluation was carried out on all the sections. To quantify the lesions in the foetal viscera and placenta, the number and size of necrotic foci, as well as the total area of lesion in the examined tissue were calculated in HE stained sections. Necrotic lesions were chosen over inflammatory ones because the former were found in all the foetuses and placentas examined while the latter were found mainly in G2 and G3. In addition, necrotic lesions are more clearly demarcated than inflammatory ones. The number of sections studied varied according to the stage of gestation. Older foetuses had larger organs (brain and liver) and therefore more samples were evaluated in these cases: in the placenta, one section from each one of the five placentomes per placenta was studied; in the foetal liver, one section from one or two different samples; and in the foetal brain, one section from one, two or three different samples. Although lesions were found in other organs (i.e. lung, heart or skeletal muscle), brain and liver were the only ones showing lesions simultaneously in all three periods studied and were therefore the only ones to be evaluated by lesional scoring. Firstly, necrotic foci in each section were counted under the microscope. Subsequently, in order to compare between organs and animals, the total number of foci per square centimetre of studied tissue was calculated. The measurement of the total area studied (TAS) is explained in the next paragraph.

A computer-assisted morphometric analysis using imageJ software [21] was carried out to calculate the areas. Digital photomicrographs from each focus of necrosis at the placenta, liver and brain were taken at 10x magnification with a calibrated Motic 2 digital camera fitted to a BA310E Motic microscope. In the digital pictures, the perimeter of each necrotic focus was traced manually and measured automatically by the software, previously calibrated to the adequate magnification. Once every focus was measured, the average size of focus (ASF) for the placenta, liver and brain was calculated. The total area of lesion (TAL) in the placenta was calculated adding up the area of each focus found in the five placentomes studied per animal. TAL in the foetal brain and liver were similarly calculated, adding up the area of each individual lesion found in every section available from each organ.

For calculating the TAS, digital photographs of the whole section were taken with the same camera fitted to a macro tube and a focusable lens. TAS measurement was done through imageJ software as well. Instead of manually tracing the area, and in order to avoid the inclusion of empty areas into the measured area such as ventricles in the nervous organs or cutting artefacts, a thresholding detection procedure and automatic measurement was applied. Briefly, the colour picture was converted to 8-bit greyscale and the threshold was manually adjusted to detect any region containing tissue sections (grey to black in colour), excluding the empty areas (white). Then, the area of the threshold regions in the picture was automatically measured by the software, previously calibrated. When more than one section was available from the same organ (i.e. two or three samples of liver or brain from the same foetus), TAS was calculated adding up the TAS of each section.

Finally, the percentage of the section affected by lesions (%LES) resulted from the coefficient between TAL and TAS per organ. In order to compare groups, averages of these measurements (i.e. foci/cm^2^, AF and %LES) obtained in the animals of each group were calculated.

### DNA extraction and PCR for parasite detection and quantification in tissues

Genomic DNA was extracted from 50–100 mg of maternal and foetal tissue samples using the commercial Maxwell® 16 Mouse Tail DNA Purification Kit, developed for automated Maxwell® 16 System (Promega, Wisconsin, USA), following the manufacturer’s recommendations. The concentration of DNA for all samples was determined by spectrophotometry and adjusted to 50–100 ng/μL.

Parasite DNA detection was carried out by a nested-PCR adapted to a single tube from the internal transcribed spacer (ITS1) region of *N. caninum*, using the external primers TgNN1-TgNN2 and internal primers NP1–NP2 as previously described [22-24]. Each reaction was performed in a final volume of 25 μL with 5 μL of sample DNA. PCR was carried out in five samples of the placentomes and three samples from the rest of tissues, except for the heart, lung and semitendinosus muscle from foetuses or lambs, in which either one, two or three samples were analysed. To avoid cross-contamination and false positive reactions, DNA extraction, PCR sample preparation and electrophoresis were performed in separate rooms employing different sets of instruments, aerosol barrier tips and disposable gloves. Moreover, both reactions without a template and DNA samples from the foetal-control group (G4) were included in each round of DNA extraction and PCR as negative controls. Positive PCR controls with *N. caninum* genomic DNA equivalent to 10, 1 and 0.1 tachyzoites in 100 ng of sheep DNA were also included in each batch of amplifications. Ten μL aliquots of the PCR products were visualized under UV light in 1.5% agarose/ethidium bromide gel to detect the *N. caninum*-specific 247 bp amplification product.

Placenta and foetal brain and liver samples that had tested positive by nested-PCR were adjusted to 20 ng DNA/μL and the parasite load was quantified using real-time PCR. Primer pairs from the *N. caninum* Nc-5 sequence [25] were used for parasite quantification, and primers from the β-actin gene [26] were used for the quantification of host DNA. Amplification reactions were performed as described by Collantes-Fernández et al. [25], in a final volume of 20 μL using Power SYBR® Green PCR Master Mix (Applied Biosystems, Foster City¸ CA, USA), 20 pmol of each primer and 100 ng of DNA in a ABI 7300 Real Time PCR System (Applied Biosystems). The *N. caninum* tachyzoite numbers were calculated by interpolating the average Ct values on two standard curves: 1) one curve equivalent to 5 × 10^5^ to 5 × 10^−1^ tachyzoites with 10-fold serial dilutions in a solution of ovine genomic DNA; and 2) a curve of 320, 160, 80, 40, 20, 10, and 5 ng of genomic DNA for ovine DNA quantifications. Parasite numbers in tissue samples (parasite burden) was expressed as parasite number/mg ovine tissue. Standard curves for *N. caninum* and sheep DNAs showed an average slope of −3.38 and −3.34, respectively, and a R^2^ > 0.99.

### Statistical analysis

Occurrence of foetal death was analysed by the Kaplan–Meier survival method. Foetal survival curves of the infected groups were then compared by the Log-rank (Mantel-Cox) test. When statistically significant differences were found, Tukey’s Multiple Range test was applied to examine all possible pairwise comparisons at each sampling time. Differences in PCR detection of parasite DNA were evaluated using the *χ*^*2*^ or Fisher exact F-test. Differences in parasite burdens and histological scoring were analysed using the non-parametric Kruskal–Wallis test followed by Dunn’s test for comparisons between groups, and the Mann–Whitney test for pairwise comparisons. Statistical significance for all analysis was established at *P* < 0.05. For statistical analysis, sentinel animals (G4) were mixed and considered as a unique control group. All statistical analyses were carried out using GraphPad Prism 5.0 software.

## Results

### Clinical observations

Foetal death was detected by US in all ewes (100%) from groups G1 (infection at 40 dg) and G2 (infection at 90 dg), although foetal death occurred significantly earlier in G1 (19–21 dpi) compared to G2 (34–48 dpi) (*P* < 0.001). In contrast, all ewes from G3 gave birth to a total of nine viable lambs between 142 and 155 dg. Three of 9 lambs were born prematurely prior to day 145, and exhibited weakness, recumbency and unresponsiveness to external stimuli. Foetal death was not detected in control groups; dams from the pregnancy control group (G5) gave birth to healthy lambs between 147 and 150 dg, and foetuses from the negative control group (G4) remained alive just before the euthanasia of their dams on day 20 pi (day 40 group), 40 pi (day 90 group) and at parturition (day 120 group).

### *Neospora*-specific IgG responses

*Neospora*-specific IgG responses were analysed by IFAT in foetal fluids, precolostral sera and sera from dams collected immediately before necropsy and are summarized in Table 2. Individual values for each dam and foetus/lamb studied are detailed in Additional file 1. Seropositive titres were detected in all infected dams, ranging from 1:1600 to 1:3200 in G1, 1:3200 to 1:6400 in G2 and 1:200 to 1:1600 in G3 (Table 2). Foetal fluids from foetuses of G1 could not be collected due to the very low volume of liquid available. For G2, seropositive titres were detected in those foetuses dying from 42 dpi onwards, ranging from 1:16 to 1:64. Precolostral sera collected from lambs born after infection at day 120 of gestation (G3), specifically those three born prior day 145 of pregnancy, yielded positive titres ranging from 1:16 to 1:128 (Table 2). Specific IgG responses against parasite antigen were not detected in animals and foetuses/lambs from the two control groups (G4 and G5).

**Table 2:** **Serological results in dams and foetuses/lambs**

**Group**	**Time of necropsy** ^**a**^	**No. of dams with the stated sera titre**					**No. of foetuses/lambs with the stated FL/PCS titre**						
		**1/200**	**1/800**	**1/1600**	**1/3200**	**1/6400**	**NA**	**-**	**1/8**	**1/16**	**1/32**	**1/64**	**1/128**
G1 (day 40)	19-21 dpi			4	2		11						
G2 (day 90)	34-48 dpi				4	3	3	1	1	1	3	3	
G3 (day 120)	142-155 dg	1	3	3				4	2*	1*		1	1

^a^Necropsies were carried out when foetal dead was detected or immediately after parturition.

*Lambs prematurely born showing weakness and unresponsiveness.

dpi: days post-infection; dg: days of gestation; NA: not available; FL: foetal liquid; PCS: precolostral serum.

### Pathology and lesion quantification

#### Gross lesions

At the time of necropsy, detachment of the placenta from the uterus was evident in one animal from G1 and three animals from G2. In G3, placenta and uterus could only be recovered from three animals, while in the remaining four animals from this group placentas were too autolytic when recovered after lambing, rendering proper examination impossible.

**Figure 1:**
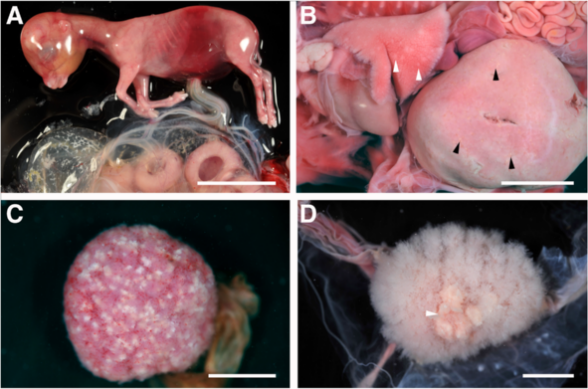
**Macroscopic lesions in foetuses and placentas. A**: haemorrhage in the abdominal region of a non-viable foetus from G1 (infection at 40 dg). Note the absence of gross lesions in the placentomes at the lower part of the image. Bar 3 cm. **B**: multiple minute white/yellowish foci of necrosis in the lung (white arrowheads) and liver (black arrowheads) in a non-viable foetus from G1 (infection at 40 dg). Bar: 1 cm. **C**: Multiple white foci of necrosis scattered in the surface of a cotyledon from a G2 placenta. Bar: 1.5 cm. **D**: Aggregation of cotyledonary villi (white arrowhead) in a placenta from G2. Bar: 1.5 cm.

In G1, all foetuses examined showed a variable degree of abdominal haemorrhage and subcutaneous oedema (Figure 1A). One foetus from this group showed multifocal white spots in the lung (1–2 mm of diameter) and liver (of miliary size) (Figure 1B). No gross lesions were visible in any placenta from this group. In groups G2 and G3 there were macroscopic lesions in the placenta. In two animals, one from each group, multifocal white foci in the cotyledons were visible (Figure 1C). In three further animals from G2 and one from G3 there were focal aggregations of the cotyledonary villi (Figure 1D). No macroscopic lesions in any of the foetuses from these two groups were found, and no foetal lesions were detected in G4 and G5.

#### Microscopic lesions

**Figure 2:**
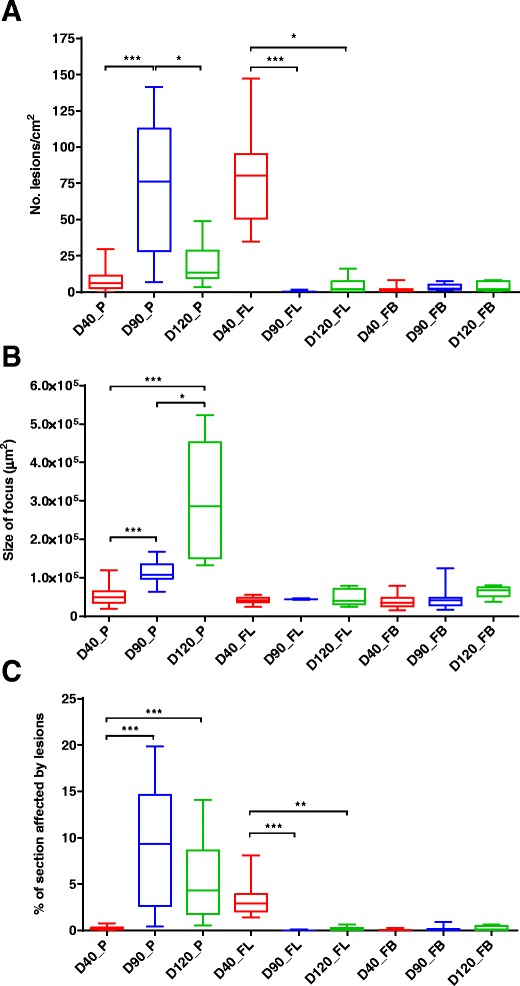
**Box-plot graphs of histological scores.** Number of lesions per centimetre square **(a)**, size of focus **(b)** and lesions rates **(c)** in placenta (P), foetal liver (FL) and foetal brain (FB) from animals inoculated with 10^6^ Nc-Spain7 tachyzoites at days 40 -G1- (D40), 90 -G2- (D90) and 120 -G3- (D120) of gestation. Box-plot graphs represent the median percentage, the lower and upper quartiles (boxes) and minimum and maximum values (whiskers). (*) indicates *P* < 0.05, (**) *P* < 0.01 and (***) *P* < 0.001 significant differences between infected groups in each tissue.

The quantification of lesion size and number from placenta as well as foetal liver and brain is shown in Table 3 and Figure 2. Individual values for each foetus or placental sample studied are detailed in Additional file 2.

**Table 3:** **Lesion quantification and PCR detection and quantification of** ***N. caninum*** **in placenta and foetal liver and brain**

**Group**	**Foetal death (dpi)**	**Placenta**				**Liver**				**Brain**			
		**Histology** ^**α**^			**PCR** ^**β**^	**Histology** ^**α**^			**PCR** ^**β**^	**Histology** ^**α**^			**PCR** ^**β**^
		**No. foci/cm** ^**2**^	**ASF (mm** ^**2**^ **)**	**%LES**		**No. foci/cm** ^**2**^	**ASF (mm** ^**2**^ **)**	**%LES**		**No. foci/cm** ^**2**^	**ASF (mm** ^**2**^ **)**	**%LES**	
G1 (day 40)	19-21	8.2^d^	0.053^d^	0.25%^d^	6/6 (7888^c^)	82.3^c^	0.041	3.37%^c^	11/11^a^ (6318^c^)	1.68	0.038	0.05%	11/11 (1697^c^)
G2 (day 90)	34-48	73.0^c^	0.113^d^	9.07%^c^	7/7 (308.5^d^)	0.2^d^	0.044	0.02%^d^	5/12^b^ (0.01^d^)	3.14	0.047	0.18%	10/12 (0.25^d^)
G3 (day 120)	Born*	18.7^d^	0.304^c^	5.74%^c^	7/7 (190.8^d^)	4.2^d^	0.047	0.16%^d^	4/9^b^ (0.01^d^)	3.50	0.063	0.25%	6/9 (0.01^d^)

*All lambs from this group gave birth to viable lambs between days 142 and 155 of gestation.

^α^Average values of histological scoring for each group.

^β^Fractions represent the number of positive animals/total number of animals checked by nested-ITS1 PCR, and figures within brackets represent the median values of parasite burden (tachyzoites/mg tissue).

^a,b^Fractions determined for positive animals followed by unlike superscripts differ significantly by Fisher’s exact test.

^c,d^Values followed by unlike superscripts differ significantly by Dunn’s test for pairwise comparisons.

dpi: days post infection when abortion occurred; ASF: Average size of focus; %LES: Percentage of section affected by lesions.

#### Placenta

**Figure 3:**
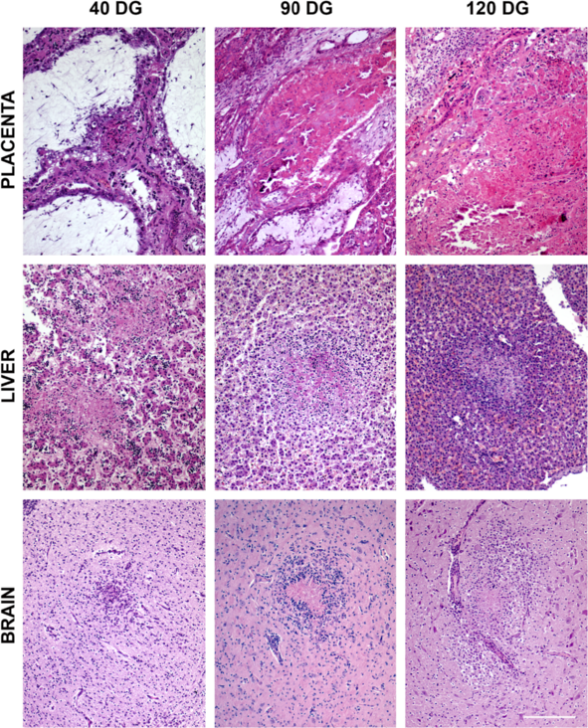
**Comparison of the characteristic microscopic lesions found in placenta and foetal liver and brain.** Pictures show the histological changes measured for the quantification of lesions. When necrotic areas and infiltration of inflammatory cells coexisted, mainly at the liver and brain of G2 and G3, only the area showing necrosis was measured. All the pictures were taken at the same magnification. Bar 200 μm.

All the placentas studied from infected animals showed multifocal non-purulent necrotic placentitis characterized by randomly distributed foci of necrosis in the placentome. The only difference between groups was the size and number of necrotic foci. In G1, the foci were well delimited, small (0.05 mm^2^ in average) and affected the caruncular septa of the placentome, causing flattening or loss of the adjacent trophoblasts. The foetal mesenchyme in the vicinity of these foci showed hypercellularity when compared with non-affected regions. Inflammation was not a prominent feature, although occasional accumulation of lymphocytes was found between caruncles and foetal villi. Placentas from G2 showed necrotic foci of similar characteristics but larger (an average of 0.1 mm^2^) than those in G1 (*P* < 0.001). They were almost exclusively located in the caruncular septa, although the largest could also affect the foetal mesenchyme. A number of these foci showed mineralization of the necrotic core area. Lesions in G3 placentas were also similar, only larger than those in previous groups (0.3 mm^2^ in average) (*P* < 0.001 for G1 and *P* < 0.05 for G2). Some of the larger foci were made by the coalescence of several, smaller ones. Similarly to the other groups, inflammatory cells were not frequently observed, but when present lymphocytes were mainly distributed along the periphery of the lesion in the foetal mesenchyme (Figure 3). When analysing the percentage of damaged area in relation to the whole tissue section, there was a significant difference between placentas from G1 (0.25%) and those from G2 (9.07%) and G3 (5.74%) (*P* < 0.001).

#### Liver

Lesions were characterized by the presence of multiple foci of coagulative necrosis randomly distributed in the hepatic parenchyma (Figure 3). Differences in lesion size and severity were observed between groups. While in foetuses from G1 lesions coalesced forming large areas of necrosis, in G2 and G3 the necrotic foci were individually well-demarcated. Inflammation was not seen in samples from G1, but there was a mild infiltration surrounding the necrotic areas formed mainly by lymphocytes. When comparing the number and size of lesions among the groups, it should be highlighted that only two foetuses from G2 had necrotic foci in the liver, while liver lesions were found in all foetuses from G1 and all but 2 in G3. There were no significant differences in the size of lesions when they were present, but the amount increased drastically in G1, which exhibited the highest percentage of affected parenchyma (*P* < 0.01).

#### Brain

The common lesion in all the groups was multifocal non-purulent necrotizing encephalitis characterized by the presence of randomly distributed glial foci. While in G1 necrosis was rarely observed, lesions in G2 and G3 were characterized by large necrotic foci surrounded by mononuclear cells, consistent with microglia and lymphocytes, forming a palisade. Besides the increase on the necrotic component, lesions in G2 and G3 were also larger than those found in G1, although differences in the total damaged area were not statistically significant (Figure 3).

#### Other organs

Foetuses from G1 showed multiple areas of necrosis randomly distributed in the lung but also in the heart and skeletal muscle (Additional file 3A). Occasionally, and mainly in the heart, these foci showed central mineralization. There was no evident infiltration related to these lesions. However, in the foetuses from G2 and G3, lesions were mainly found in the skeletal muscle, characterized by a multifocal non-purulent myositis (Additional file 3B), although occasional neutrophils could be identified among the muscular fibres. The latter were seen as unaffected, healthy fibres or exhibited degenerative changes characterized by loss of striation, hyper-eosinophilic cytoplasm and rarely mineralization. No obvious signs of fibre regeneration were found. Milder lesions were occasionally found in the heart, mainly in G3. No evident lesion was found in the lungs of G2 or G3.

No significant microscopic lesion was seen in the placenta or foetuses of G4 and G5.

### Parasite distribution and burden in placental and foetal tissues

#### Maternal tissues

*N. caninum* DNA was detected in both mesenteric and iliofemoral lymph nodes of ewes from all three infected groups. Parasite DNA was more often detected in lymph node samples from G1 compared to G2 and G3 (*P* < 0.05). 5/6 mesenteric and 4/6 iliofemoral lymph nodes from G1 harboured parasite DNA, compared to G2 (1/7 and 2/7, respectively) and G3 (2/7), although significant differences were only found between mesenteric lymph nodes from G1 and G2 (*P* < 0.05). Parasite DNA was not detected in any brain sample of dams from any group.

#### Placental and foetal tissues

**Figure 4:**
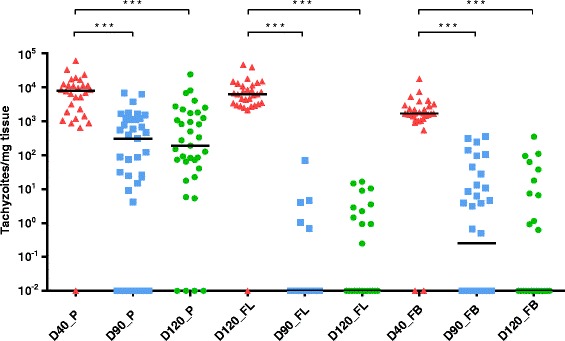
**Dot-plot graph of** ***N. caninum*** **burdens.** Parasite burdens were quantified by real-time PCR in placenta (P) and foetal liver (FL) and brain (FB) from animals inoculated with 10^6^ Nc-Spain7 tachyzoites at days 40 -G1- (D40), 90 -G2- (D90) and 120 -G3- (D120) of gestation. Each dot represents individual values of parasite burden (number of parasites per mg of host tissue), and medians are represented as horizontal lines. Taking into account that the *N. caninum* detection limit by real-time PCR is 0.1 parasites, negative samples (0 parasites) were represented on the log scale as < 0.1 (i.e. 10^−2^). (***) indicates *P* < 0.001 significant differences between infected groups in each tissue.

*Neospora* DNA was widely detected in placentomes from all ewes in the three infected groups (Additional file 4), with 97% of the placentome samples analysed being positive in G1 (29/30), 83% in G2 (29/35) and 89% in G3 (31/35), with no statistical significances between them. Nevertheless, the mean parasite burden, measured as number of tachyzoites per mg of tissue, was significantly higher in placentomes from G1 compared to both G2 and G3 (*P* < 0.001) (Figure 4, Table 3).

Regarding foetal tissues, the highest percentage of detection was observed in the brain, liver, heart and skeletal muscle of foetuses from G1 (100%, 11/11), whereas the lowest corresponded to the liver and heart of foetuses from G2 (41.6%, 5/12). In addition, significant differences were observed between G1 and G2 when comparing detection percentage in liver (*P* < 0.01), heart (*P* < 0.01) and skeletal muscle (*P* < 0.05), and also between G1 and G3 for liver samples (*P* < 0.05). Moreover, parasite burdens were significantly higher in brain and liver from G1 compared to G2 and G3 (*P* < 0.001) (Figure 4). When comparing parasite burdens in foetuses from G3, those which were prematurely born before day 145 showed a statistically higher parasite burden in both foetal brain and liver compared to the 6 remaining foetuses born after day 145 (*P* < 0.01) (data not shown). As expected, all placental and foetal samples from G4 and G5 were negative.

## Discussion

Whereas *N. caninum* is established as one of the most important infectious causes of abortion in cattle worldwide [1], its epidemiological, clinical and economic relevance for reproductive failure in small ruminants has remained elusive. However recent studies in small ruminants suggest that neosporosis may be a more important cause of reproductive disorders than it has traditionally been considered, at least in certain scenarios. Recent serological surveys have shown high prevalences in asymptomatic sheep and goats from some flocks [1] and evidence has accumulated that points towards *N. caninum* as a cause of natural abortion and reproductive failure in sheep and goats [6-8,27].

The aim of this study was to characterize the clinical, parasitological and lesional parameters in experimental ovine neosporosis at early (40 dg), mid (90 dg) and late (120 dg) stages of pregnancy, to better understand the pathogenesis of *N. caninum* infection. To our knowledge, this is the first study in which all three periods of gestation have been tested within the same experiment under the same conditions (isolate, breed, dose and route of administration). In addition, and in the absence of broadly accepted international guidelines on models for ruminant neosporosis [17], there is a need to establish a suitable and economically viable experimental model for the disease. With this in mind, sheep have been evaluated as suitable experimental model for neosporosis in this study.

Experimental infection was carried out using the *N. caninum* Nc-Spain7 isolate [18], which is a well-characterized highly virulent isolate. Indeed, this isolate caused 100% foetal death upon experimental infection of pregnant cows during the first trimester of gestation [22,28]. The intravenous route of inoculation used in this study was chosen so as to mirror parasitaemia produced in natural infections [29]. Finally, the inoculation dose, 10^6^ tachyzoites, was a 1:10 dilution of that commonly used in cattle (i.e. 10^7^ tachyzoites) [30-33], considering the weight difference between cattle and sheep (aprox. 10:1).

Clinical observations revealed that infection during the first (G1) and second (G2) term of gestation produced foetal death in all ewes, although it happened significantly earlier in the G1. On the other hand, infection during the last term of gestation (G3) resulted in the birth of viable lambs, although some of them were born prematurely and showed evident weakness that, most probably, would have compromised their viability. Infection during the first period of gestation (G1) showed similar results to those reported in previous experiments carried out at early gestation in both cattle [28,30,34] and sheep [9], where foetal death and abortion were observed. In the current study, lesions and parasite burden found in both dams and foetuses confirm that the parasite readily disseminates in pregnant sheep, crosses the placenta and subsequently induces foetal damage. In this group, high parasite burdens were found in all studied placentomes, but only mild lesions were observed, which might be attributed to the short period of time that occurred between infection and abortion (19–21 days), thus limiting the development of histological lesions. The only placenta studied in previous experimental infections at 45 dg also showed mild lesions [9]. Interestingly, these findings are in clear contrast with the widespread necrosis and inflammation reported in placenta from cattle infected at 70 dg [22,35,36], which suggests that there could be differences between cattle and sheep regarding the initial infection dynamics. It has already been suggested that the lack of mature immune response at this stage of foetal development would allow the parasite to cross the placenta and colonise foetal viscera and multiply with ease [22,36]. Indeed, the highest frequency of detection and parasite burden in the current study were detected in foetuses from this group (G1), where *N. caninum* was widely detected in the brain, liver, heart and semitendinosus muscle of all foetuses. There was a good correlation between parasite burden and frequency of lesions in the foetal liver, which most probably represents the gateway for the parasite to invade the foetus through the umbilical vein. This correlation did not occur in the brain, where there was a high parasite burden, though not as high as in the liver, but the histological lesions were much less frequent. While the reason for this difference is not clear, it is known that antigen levels required to produce lesions in the immune privileged brain are, at least, ten times higher than those needed in other organs, and inflammatory reactions require more time to develop [37]. Thus, the low frequency of lesions in the brain at this stage may be caused by the short period of time since infection. The contrast between mild lesions in the placenta and severe multifocal necrosis in the foetal liver may suggest that the abortion at this stage of gestation is consequence of the foetal lesions, similarly as suggested to occur in bovine neosporosis at early gestation [22,36].

Previous studies addressing the infection in ewes during mid-gestation (mainly at day 90) have reported a variety of results, which could be attributed to the different time points chosen for infection as well as the isolate, dose, breed and the employed route of administration [9,11,12,38]. Nevertheless, all of them reported high rates, of even 100%, of abortion. Conversely, the most commonly reported outcome in cattle after experimental infection at mid-gestation is the delivery of live, although congenitally infected, calves [39-41]. However, abortion has also been reported [31,42] and it is actually at this stage when most of the abortions in natural infection occur [2,17]. Considering the findings from the current study and previous experimental infections, it seems that infection of sheep at mid-gestation generally results in more dramatic consequences than in cattle, as most of the ewes aborted or, less frequently, produced weak lambs. Although these differences may be related to the species, similarly to the discrepancy between sheep and cattle regarding the placental damage at early gestation, it is also possible that the doses used in this and previous experiments (even up to 10^8^) have been excessively aggressive for the infected sheep. This suggestion is supported by the fact that previous studies employing lower doses obtained more variable outcomes, ranging from few aborted foetuses to birth of weak or healthy lambs [11,12]. Nevertheless, information concerning parasite dose and isolate virulence is limited. Hence, in order to address the standardization of a pregnant model in sheep, further experiments would be desirable to compare outcomes from different inoculation doses and various *N. caninum* isolates. Our study confirmed that around 90 dg the foetal immune system is undergoing development according to specific antibodies detected in foetal fluids, and the lower parasite burden found in this group compared to G1 (day 40). This suggests a partial, yet insufficient, control of the infection.

The finding of more severe lesions in the placenta found at G2 despite the lower parasite burden suggests that the inflammatory response developed in this organ has been effective in controlling the parasite, maybe because of the longer period of time elapsed since infection, but has also damaged the organ as a “bystander effect”. Indeed, the highest ewe serum titres were found in this group, probably due to the longer time before abortion occurred. In foetal samples, parasite detection (both frequency and burden) were lower in comparison to G1, but the frequency of lesions in the brain and skeletal muscles were higher. This, together with the shift from necrotic to inflammatory characteristic of the lesions, was most likely also a consequence of the maturation of foetal immune response. The dramatically smaller number of lesions in the liver compared to G1 may be consequence of the longer period of time elapsed since infection. The liver is probably the first foetal organ reached by the parasite through the umbilical vein, so the three additional weeks compared to G1 may have given the organ time to clear the lesions, as it has been previously observed [9].

With regard to infection at late pregnancy (G3), all ewes delivered live lambs, although three of them were born before day 145 and showed weakness and unresponsiveness. These results are in accordance with those previously described for both pregnant cattle [43-45] and sheep [12], in which the delivery of live, but congenitally infected, calves or lambs with no obvious clinical signs is the most common consequence of experimental infection during the third term of gestation. However, although infection of the placenta and foetuses (i.e. transplacental infection) occurred in all sheep from this group, the three lambs prematurely born showed both higher parasite burden and number of lesions in brain and liver compared to the other six lambs born after day 145. In addition, parasites could only be detected in the lymph nodes from the dams that delivered these three premature animals. In the remaining lambs, parasite detection was higher when compared to that observed in G2, except for one of the twin lambs delivered at day 155 of gestation, which was completely negative by PCR. This could be attributed to the difference in time at necropsy between the two groups, since lambs from G3 were studied two weeks earlier than those from G2, giving therefore less time to the immune response, already mature in both cases, to control the infection. Sera from G3 lambs collected before suckling showed a range of titres from negative to 1:128. Four of the 6 lambs born apparently healthy after day 145 of gestation were seronegative. Despite this, 3 of these 4 lambs were infected as proven by PCR. Moreover, as lambs were culled soon after birth, we do not know what would have happen thereafter, and can only hypothesise that those lambs with negative sera titres would have survived as persistently infected animals, whereas those prematurely born with high parasite detection and showing weakness and recumbency may have not survived long after delivery, as they were too weak to be nourished by the mother.

Finally, the quantification of histological lesions in the placenta and foetuses has shown to be very useful in adding to the interpretation of the results from this experiment. In a similar approach, Buxton et al. [14] described how the number of necrotic foci in the placenta increased with the time post-infection up to a maximum, around 40 days pi, from where the organ recovered and the number of foci decreased sharply. These observations correlates with our results, suggesting that: a) there is a relation between the time post-infection and the severity of the lesions and b) lesions may regress in the affected organs. The latter has been described to occur in the placenta [14] and, according to the present results, also in the foetal liver, but not in the foetal brain.

In the light of the results from this study, we suggest that the experimental infection of sheep had similar outcomes, in term of abortions and birth of weak or clinically healthy lambs, to those described in natural bovine or ovine neosporosis. Despite a number of differences, which trigger further investigations of ovine neosporosis, the use of an ovine model seems reasonably valid for assessments of both, vaccines and drug candidates for bovine neosporosis. In addition, this model is uniquely suited to investigate the pathogenesis of ovine neosporosis, a disease with increasing relevance. Further studies would be desirable in order to achieve a better and well-established *N. caninum* infection model in sheep [17].

## Additional files

Additional file 1:
**Individual serological titres in dams and foetuses/lambs at the time of necropsy.** ^a^Necropsies were carried out when foetal dead was detected or immediately after parturition. *Lamb prematurely born showing weakness and unresponsiveness. dpi: days post-infection; dg: days of gestation; NA: not available; FL: foetal liquid; PCS: precolostral serum. Additional file 2:
**Individual quantification of lesions in the placenta, foetal liver and brain.** ^a^Necropsies were carried out when foetal dead was detected or immediately after parturition. *Lamb prematurely born showing weakness and unresponsiveness. dpi: days post-infection; dg: days of gestation; NA: not available. Additional file 3:
**Sections of skeletal muscle from non-viable foetuses.** A: large area of coagulative necrosis in the skeletal muscle of a foetus from G1. Note the scarcity of inflammatory cells related to this lesion. B: diffuse infiltration of mixed inflammatory cells among muscular fibres. With this lesions, note the coexistence of degenerated (arrows) and preserved (arrowheads) fibres. Both pictures were taken at the same magnification. Bar: 200 μm. Additional file 4:
**Individual frequency of parasite DNA detection.** ^a^Necropsies were carried out when foetal dead was detected or immediately after parturition. *Lamb prematurely born showing weakness and unresponsiveness. dpi: days post-infection; dg: days of gestation; MLN: mesenteric lymph node; ULN: uterine lymph node; FL: foetal liquid; PCS: precolostral serum; STM semitendinosus muscle; plus (+++, ++, +) and minus (−) signs represent PCR detection in >67%, 66-34%, <33% and 0% of samples analysed, respectively.
