# Supplementary material for: Influence of the gestational stage on the clinical course, lesional development and parasite distribution in experimental ovine neosporosis
Source: Vet Res. 2015 Mar 3;46:19. doi: 10.1186/s13567-014-0139-y (PMC4346111; doi:10.1186/s13567-014-0139-y)
Supplement: Additional file 1: — Individual serological titres in dams and foetuses/lambs at the time of necropsy. aNecropsies were carried out when foetal dead was detected or immediately after parturition. *Lamb prematurely born showing weakness and unresponsiveness. dpi: days post-infection; dg: days of gestation; NA: not available; FL: foetal liquid; PCS: precolostral serum. [file 13567_2014_139_MOESM1_ESM.docx]

**Additional file 1 Individual serological titres in dams and foetuses/lambs** **at the time of necropsy.**

| **Group** | **Ewe ref.** | **Time of necropsy^a^** | **Dams sera titre** |  | **Foetus/Lambs ref.** | **FL or PCS titre** |
| --- | --- | --- | --- | --- | --- | --- |
| **G1**  **(day 40)** | **E412** | 19 dpi | 3200 |  | **F412** | NA |
|  | **E402** | 20 dpi | 1600 |  | **F402-1** | NA |
|  |  |  |  |  | **F402-2** | NA |
|  | **E009** | 21 dpi | 1600 |  | **F009-1** | NA |
|  |  |  |  |  | **F009-2** | NA |
|  | **E411** | 21 dpi | 1600 |  | **F411** | NA |
|  | **E533** | 21 dpi | 3200 |  | **F533-1** | NA |
|  |  |  |  |  | **F533-2** | NA |
|  | **E537** | 21 dpi | 1600 |  | **F537-1** | NA |
|  |  |  |  |  | **F537-2** | NA |
|  |  |  |  |  | **F537-3** | NA |
| **G2**  **(day 90)** | **E023** | 34 dpi | 3200 |  | **F023** | - |
|  | **E010** | 36 dpi | 6400 |  | **FE010-1** | NA |
|  |  |  |  |  | **FE010-2** | - |
|  | **E011** | 42 dpi | 6400 |  | **F011-1** | 32 |
|  |  |  |  |  | **F011-2** | 32 |
|  | **E016** | 42 dpi | 3200 |  | **F016-1** | 32 |
|  |  |  |  |  | **F016-2** | 64 |
|  | **E021** | 42 dpi | 6400 |  | **F021** | 64 |
|  | **E002** | 48 dpi | 3200 |  | **F002-1** | 64 |
|  |  |  |  |  | **F002-2** | 16 |
|  | **E026** | 48 dpi | 3200 |  | **F026-1** | 8 |
|  |  |  |  |  | **F026-2** | NA |
| **G3**  **(day 120)** | **E382** | 142 dg | 1600 |  | **L382*** | 8 |
|  | **E522** | 143 dg | 800 |  | **L522*** | 8 |
|  | **E030** | 144 dg | 800 |  | **L030*** | 16 |
|  | **E380** | 145 dg | 1600 |  | **L380-1** | - |
|  |  |  |  |  | **L380-2** | 64 |
|  | **E014** | 149 dg | 1600 |  | **L014** | 128 |
|  | **E441** | 155 dg | 200 |  | **L441-1** | - |
|  |  |  |  |  | **L441-2** | - |
|  | **E523** | 155 dg | 800 |  | **L523** | - |

^a^ necropsies were carried out when foetal dead was detected or immediately after parturition.

^*^ lamb prematurely born showing weakness and unresponsiveness.

dpi: days post-infection; dg: days of gestation; NA: not available; FL: foetal liquid; PCS: precolostral serum.
