# Supplementary material for: Influence of the gestational stage on the clinical course, lesional development and parasite distribution in experimental ovine neosporosis
Source: Vet Res. 2015 Mar 3;46:19. doi: 10.1186/s13567-014-0139-y (PMC4346111; doi:10.1186/s13567-014-0139-y)
Supplement: Additional file 2: — Individual quantification of lesions in the placenta, foetal liver and brain. aNecropsies were carried out when foetal dead was detected or immediately after parturition. *Lamb prematurely born showing weakness and unresponsiveness. dpi: days post-infection; dg: days of gestation; NA: not available. [file 13567_2014_139_MOESM2_ESM.docx]

**Additional file 2 Individual quantification of lesions in the placenta, foetal liver and brain**.

| **Group** | **Foetus or lamb reference** | **Time of necropsy^a^** |  | **Placentome** | | |  | **Foetal liver** | | |  | **Foetal brain** | | |
| --- | --- | --- | --- | --- | --- | --- | --- | --- | --- | --- | --- | --- | --- | --- |
|  |  |  |  | No. foci per cm^2^ | Size (10^3^ µm^2^) | Tissue affected (‰) |  | No. foci per cm^2^ | Size  (10^3^ µm^2^) | Tissue affected (‰) |  | No. foci per cm^2^ | Size  (10^3^ µm^2^) | Tissue affected (‰) |
| **G1**  **(day 40)** | **F412** | 19 dpi |  | 4.38 | 61.685 | 2.88 |  | 147.22 | 54.854 | 80.755 |  | 0.60 | 43.567 | 0.259 |
|  | **F402-1** | 20 dpi |  | NA | NA | NA |  | 46.93 | 43.821 | 20.563 |  | - | - | - |
|  | **F402-2** | 20 dpi |  |  |  |  |  | 67.64 | 41.063 | 27.774 |  | - | - | - |
|  | **F009-1** | 21 dpi |  | 6.91 | 54.904 | 2.82 |  | 78.69 | 46.317 | 36.448 |  | 0.52 | 26.825 | 0.140 |
|  | **F009-2** | 21 dpi |  |  |  |  |  | 86.52 | 50.082 | 43.332 |  | - | - | - |
|  | **F411** | 21 dpi |  | 10.29 | 40.579 | 2.38 |  | 34.82 | 39.906 | 13.895 |  | 2.17 | 24.676 | 0.535 |
|  | **F533-1** | 21 dpi |  | 17.86 | 44.904 | 3.90 |  | 86.15 | 33.718 | 29.049 |  | 1.64 | 48.959 | 0.804 |
|  | **F533-2** | 21 dpi |  |  |  |  |  | 108.79 | 35.997 | 39.162 |  | 1.08 | 15.056 | 0.163 |
|  | **F537-1** | 21 dpi |  | 1.45 | 27.794 | 0.51 |  | 95.17 | 24.571 | 23.383 |  | 8.21 | 29.132 | 1.314 |
|  | **F537-2** | 21 dpi |  |  |  |  |  | 50.64 | 38.060 | 19.273 |  | 0.80 | 40.909 | 0.327 |
|  | **F537-3** | 21 dpi |  |  |  |  |  | 80.42 | 47.028 | 37.819 |  | 3.51 | 78.777 | 2.764 |
| **G2**  **(day 90)** | **F023** | 34 dpi |  | NA | NA | NA |  | - | - | - |  | 5.13 | 42.068 | 2.158 |
|  | **FE010-1** | 36 dpi |  | 61.43 | 102.478 | 64.01 |  | - | - | - |  | 5.61 | 28.237 | 1.584 |
|  | **FE010-2** | 36 dpi |  |  |  |  |  | NA | NA | NA |  | 1.31 | 16.048 | 0.281 |
|  | **F011-1** | 42 dpi |  | NA | NA | NA |  | - | - | - |  | 2.28 | 39.655 | 0.905 |
|  | **F011-2** | 42 dpi |  |  |  |  |  | 0.50 | 45.948 | 0.463 |  | 2.74 | 47.658 | 1.307 |
|  | **F016-1** | 42 dpi |  | 102.39 | 144.984 | 149.58 |  | NA | NA | NA |  | 2.04 | 41.021 | 0.836 |
|  | **F016-2** | 42 dpi |  |  |  |  |  | NA | NA | NA |  | 2.57 | 25.760 | 0.663 |
|  | **F021** | 42 dpi |  | 109.43 | 122.719 | 133.09 |  | - | - | - |  | 2.45 | 81.897 | 2.009 |
|  | **F002-1** | 48 dpi |  | 18.58 | 80.809 | 16.31 |  | - | - | - |  | 1.21 | 32.636 | 0.396 |
|  | **F002-2** | 48 dpi |  |  |  |  |  | 1.67 | 42.564 | 1.419 |  | 1.67 | 124.171 | 9.485 |
|  | **F026-1** | 48 dpi |  | NA | NA | NA |  | - | - | - |  | 1.56 | 46.987 | 0.731 |
|  | **F026-2** | 48 dpi |  |  |  |  |  | - | - | - |  | NA | NA | NA |
| **G3**  **(day 120)** | **L382*** | 142 dg |  | NA | NA | NA |  | 2.39 | 34.022 | 0.813 |  | 6.01 | 74.708 | 4.493 |
|  | **L522*** | 143 dg |  | NA | NA | NA |  | 16.22 | 40.784 | 6.614 |  | 7.99 | 79.973 | 6.387 |
|  | **L030*** | 144 dg |  | NA | NA | NA |  | 8.13 | 38.292 | 3.113 |  | 7.90 | 68.132 | 5.382 |
|  | **L380-1** | 145 dg |  | 10.16 | 139.443 | 14.78 |  | 5.07 | 24.580 | 1.245 |  | 2.49 | 67.187 | 1.671 |
|  | **L380-2** | 145 dg |  |  |  |  |  | NA | NA | NA |  | NA | NA | NA |
|  | **L014** | 149 dg |  | 10.98 | 415.904 | 53.96 |  | 0.54 | 79.163 | 0.430 |  | 1.03 | 60.615 | 0.627 |
|  | **L441-1** | 155 dg |  | NA | NA | NA |  | - | - | - |  | - | - | - |
|  | **L441-2** | 155 dg |  |  |  |  |  | - | - | - |  | 0.90 | 52.187 | 0.471 |
|  | **L523** | 155 dg |  | 43.07 | 290.912 | 119.78 |  | 1.68 | 67.918 | 1.143 |  | 1.67 | 37.633 | 0.626 |

^a^ necropsies were carried out when foetal dead was detected or immediately after parturition.

^*^ lamb prematurely born showing weakness and unresponsiveness.

dpi: days post-infection; dg: days of gestation; NA: not available.
