# Supplementary figures and images for: Influence of the gestational stage on the clinical course, lesional development and parasite distribution in experimental ovine neosporosis
Source: Vet Res. 2015 Mar 3;46:19. doi: 10.1186/s13567-014-0139-y (PMC4346111; doi:10.1186/s13567-014-0139-y)

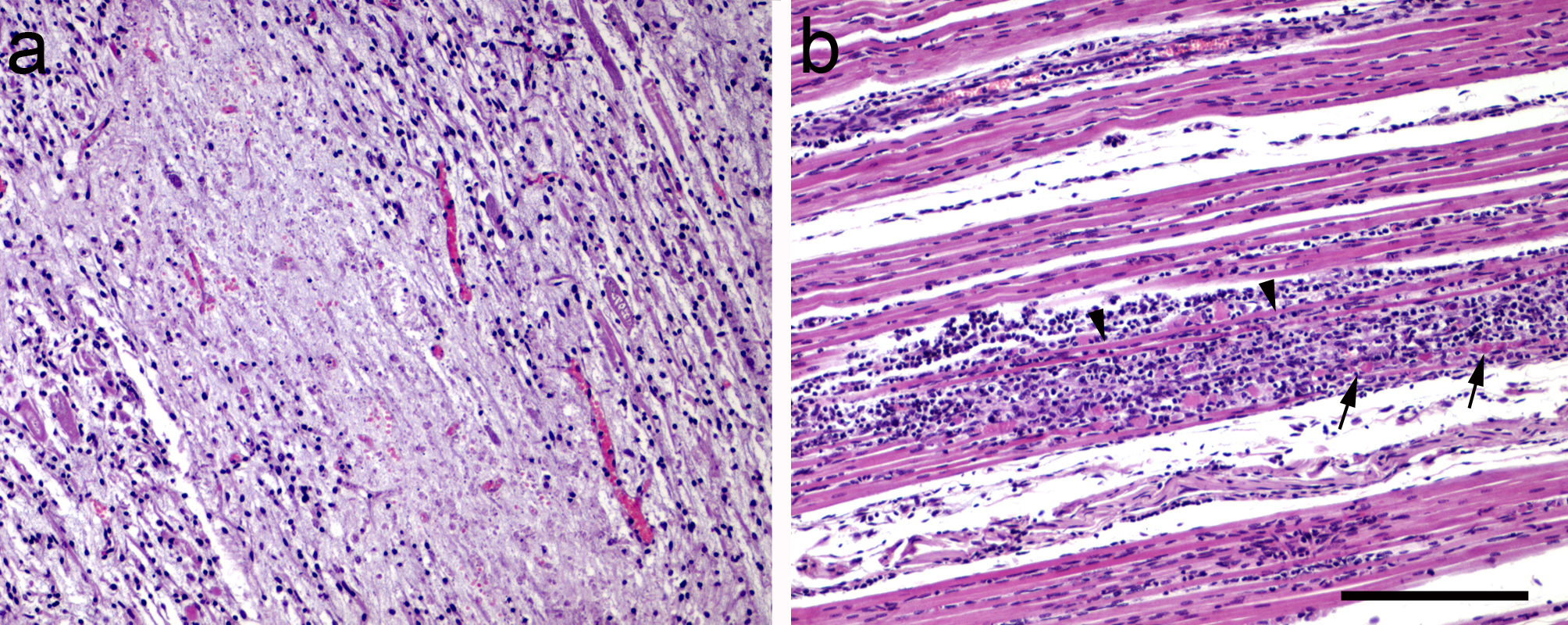

Supplement: Additional file 3: — Sections of skeletal muscle from non-viable foetuses. A: large area of coagulative necrosis in the skeletal muscle of a foetus from G1. Note the scarcity of inflammatory cells related to this lesion. B: diffuse infiltration of mixed inflammatory cells among muscular fibres. With this lesions, note the coexistence of degenerated (arrows) and preserved (arrowheads) fibres. Both pictures were taken at the same magnification. Bar: 200 μm. [file 13567_2014_139_MOESM3_ESM.jpeg]
