# Supplementary material for: Influence of the gestational stage on the clinical course, lesional development and parasite distribution in experimental ovine neosporosis
Source: Vet Res. 2015 Mar 3;46:19. doi: 10.1186/s13567-014-0139-y (PMC4346111; doi:10.1186/s13567-014-0139-y)
Supplement: Additional file 4: — Individual frequency of parasite DNA detection. aNecropsies were carried out when foetal dead was detected or immediately after parturition. *Lamb prematurely born showing weakness and unresponsiveness. dpi: days post-infection; dg: days of gestation; MLN: mesenteric lymph node; ULN: uterine lymph node; FL: foetal liquid; PCS: precolostral serum; STM semitendinosus muscle; plus (+++, ++, +) and minus (−) signs represent PCR detection in >67%, 66-34%, <33% and 0% of samples analysed, respectively. [file 13567_2014_139_MOESM4_ESM.docx]

**Additional file 4 Individual frequency of parasite DNA detection**.

| **Group** | **Foetus or Lamb reference** | **Time of necropsy^a^** |  | **Maternal tissues** | | |  | | **Foetal tissues** | | | | |
| --- | --- | --- | --- | --- | --- | --- | --- | --- | --- | --- | --- | --- | --- |
|  |  |  |  | Placent. | MLN | ULN |  | Brain | | Liver | Heart | Lung | STM |
| **G1**  **(day 40)** | **F412** | 19 dpi |  | +++ | +++ | +++ |  | +++ | | +++ | +++ | +++ | +++ |
|  | **F402-1** | 20 dpi |  | +++ | +++ | - |  | +++ | | +++ | +++ | +++ | +++ |
|  | **F402-2** | 20 dpi |  |  |  |  |  | +++ | | +++ | +++ | +++ | +++ |
|  | **F009-1** | 21 dpi |  | +++ | - | - |  | +++ | | +++ | +++ | +++ | +++ |
|  | **F009-2** | 21 dpi |  |  |  |  |  | +++ | | +++ | +++ | - | +++ |
|  | **F411** | 21 dpi |  | +++ | ++ | + |  | +++ | | ++ | +++ | +++ | +++ |
|  | **F533-1** | 21 dpi |  | +++ | ++ | +++ |  | +++ | | +++ | +++ | +++ | +++ |
|  | **F533-2** | 21 dpi |  |  |  |  |  | + | | +++ | +++ | - | +++ |
|  | **F537-1** | 21 dpi |  | +++ | ++ | +++ |  | +++ | | +++ | +++ | - | +++ |
|  | **F537-2** | 21 dpi |  |  |  |  |  | +++ | | +++ | +++ | - | +++ |
|  | **F537-3** | 21 dpi |  |  |  |  |  | +++ | | +++ | +++ | ++ | +++ |
| **G2**  **(day 90)** | **F023** | 34 dpi |  | +++ | + | ++ |  | +++ | | + | ++ | - | + |
|  | **FE010-1** | 36 dpi |  | +++ | - | - |  | ++ | | + | - | + | + |
|  | **FE010-2** | 36 dpi |  |  |  |  |  | +++ | | - | - | + | - |
|  | **F011-1** | 42 dpi |  | +++ | - | - |  | + | | - | - | - | ++ |
|  | **F011-2** | 42 dpi |  |  |  |  |  | + | | + | + | ++ | - |
|  | **F016-1** | 42 dpi |  | +++ | - | - |  | ++ | | - | - | - | ++ |
|  | **F016-2** | 42 dpi |  |  |  |  |  | +++ | | - | ++ | ++ | + |
|  | **F021** | 42 dpi |  | +++ | - | ++ |  | + | | - | - | - | - |
|  | **F002-1** | 48 dpi |  | +++ | - | - |  | - | | - | + | +++ | - |
|  | **F002-2** | 48 dpi |  |  |  |  |  | + | | + | + | +++ | + |
|  | **F026-1** | 48 dpi |  | ++ | - | - |  | + | | + | - | +++ | - |
|  | **F026-2** | 48 dpi |  |  |  |  |  | - | | - | - | +++ | - |
| **G3**  **(day 120)** | **L382*** | 142 dg |  | +++ | - | + |  | +++ | | - | + | + | + |
|  | **L522*** | 143 dg |  | +++ | ++ | - |  | +++ | | +++ | +++ | +++ | +++ |
|  | **L030*** | 144 dg |  | +++ | + | ++ |  | +++ | | +++ | +++ | +++ | +++ |
|  | **L380-1** | 145 dg |  | +++ | - | - |  | - | | ++ | +++ | +++ | +++ |
|  | **L380-2** | 145 dg |  |  |  |  |  | +++ | | +++ | ++ | +++ | +++ |
|  | **L014** | 149 dg |  | +++ | - | - |  | - | | - | +++ | +++ | +++ |
|  | **L441-1** | 155 dg |  | ++ | - | - |  | - | | - | - | - | - |
|  | **L441-2** | 155 dg |  |  |  |  |  | + | | - | ++ | +++ | ++ |
|  | **L523** | 155 dg |  | +++ | - | - |  | ++ | | - | - | - | + |

^a^ necropsies were carried out when foetal dead was detected or immediately after parturition.

^*^ lamb prematurely born showing weakness and unresponsiveness.

dpi: days post-infection; dg: days of gestation; MLN: mesenteric lymph node; ULN: uterine lymph node; FL: foetal liquid; PCS: precolostral serum; STM semitendinosus muscle; plus (+++, ++, +) and minus (-) signs represent PCR detection in >67%, 66-34%, <33% and 0% of samples analysed, respectively.
